# Dexmedetomidine improves the circulatory dysfunction of the glymphatic system induced by sevoflurane through the PI3K/AKT/ΔFosB/AQP4 pathway in young mice

**DOI:** 10.1038/s41419-024-06845-w

**Published:** 2024-06-25

**Authors:** Shuying Wang, Xiaojin Yu, Lili Cheng, Weishu Ren, Gehua Wen, Xue Wu, Haoyang Lou, Xinghua Ren, Lei Lu, Anca Hermenean, Jun Yao, Baoman Li, Yan Lu, Xu Wu

**Affiliations:** 1 School of Forensic Medicine, China Medical University, Shenyang, China; 2 Department of Anaesthesiology, The First Hospital of China Medical University, Shenyang, China; 3 Department of Anaesthesiology, Affiliated Shengjing Hospital of China Medical University, Shenyang, China; 4 Department of pediatrics Neonatology, University of Chicago, Chicago, IL 60615 USA; 5 Faculty of Medicine, Vasile Goldis Western University of Arad, Arad, Romania; 6 Liaoning Province Key Laboratory of Forensic Bio-evidence Sciences, Shenyang, China; 7 China Medical University Center of Forensic Investigation, Shenyang, China; 8 Key Laboratory of Health Ministry in Congenital Malformation, Affiliated Shengjing Hospital of China Medical University, Shenyang, China

**Keywords:** Preclinical research, Neuronal development, Developmental neurogenesis, Cognitive control

## Abstract

Multiple sevoflurane exposures may damage the developing brain. The neuroprotective function of dexmedetomidine has been widely confirmed in animal experiments and human studies. However, the effect of dexmedetomidine on the glymphatic system has not been clearly studied. We hypothesized that dexmedetomidine could alleviate sevoflurane-induced circulatory dysfunction of the glymphatic system in young mice. Six-day-old C57BL/6 mice were exposed to 3% sevoflurane for 2 h daily, continuously for 3 days. Intraperitoneal injection of either normal saline or dexmedetomidine was administered before every anaesthesia. Meanwhile the circulatory function of glymphatic system was detected by tracer injection at P8 and P32. On P30-P32, behavior tests including open field test, novel object recognition test, and Y-maze test were conducted. Primary astrocyte cultures were established and treated with the PI3K activator 740Y-P, dexmedetomidine, and small interfering RNA (siRNA) to silence ΔFosB. We propose for the first time that multiple exposure to sevoflurane induces circulatory dysfunction of the glymphatic system in young mice. Dexmedetomidine improves the circulatory capacity of the glymphatic system in young mice following repeated exposure to sevoflurane through the PI3K/AKT/ΔFosB/AQP4 signaling pathway, and enhances their long-term learning and working memory abilities.

## Introduction

More and more children in early childhood are receiving effective interventions, painless examinations, and surgical treatments for various diseases. However, along with these advancements, there comes the potential for repeated exposure to anesthesia. In 2016, the U.S. Food and Drug Administration (FDA) issued a safety warning stating that repeated exposure to general anesthesia drugs in children under the age of 3 may affect the brain development of the children [1]. Subsequently, clinical studies such as GAS [2, 3] and PANDA [4] have indicated that a single short-term anesthesia exposure does not have long-term developmental effects on children. However, these studies do not account for children who have undergone multiple and prolonged anesthesia exposures. The MASK study [5], which conducted statistical analysis on children who underwent single and multiple anesthesia exposures, found that while children with multiple anesthesia exposures did not show intellectual developmental abnormalities, they exhibited impaired fine motor skills and abnormal reading processing speed. Therefore, effective interventions need to be further studied.

The high metabolic activity of neurons in the brain requires the rapid elimination of metabolic byproducts to maintain a normal physiological environment. However, the brain lack lymphoid tissue. The glymphatic system facilitate solute exchange from the brain parenchyma and interstitial fluid, relying on the perivascular space as an anatomical structure [6, 7]. Sevoflurane, due to its pleasant odor and low respiratory irritability, is widely used in pediatric clinical anesthesia [8]. Studies have shown that different anesthetic agents have varying effects on the circulatory function of the glymphatic system [9]. Therefore, it is hypothesized that repeated exposure to sevoflurane likely impairs the circulatory function of the glymphatic system in young mice, leading to the accumulation of metabolic waste products.

ΔFosB is a member of the Fos transcription factor family and is known to accumulate with repeated stimulation. It has been most studied in animal models of drug addiction and chronic stress [10, 11]. Therefore, we hypothesize that repeated exposure to sevoflurane in young mice may induce an acute elevation of ΔFosB. Using the JASPAR database, we predicted a binding site between ΔFosB and AQP4 promoter. The expression level and polarity arrangement of AQP4 are positively correlated with the circulatory function of the glymphatic system [12, 13]. This further confirms the potential damage to the circulatory capacity of the glymphatic system caused by repeated exposure to sevoflurane.

Dexmedetomidine exerts neuroprotective effects through various pathways, including anti-inflammatory and anti-apoptotic mechanisms [14, 15]. Recent studies have shown that dexmedetomidine can enhance the circulatory capacity of the brain’s glymphatic system [16, 17], although the specific regulatory mechanisms are not yet clear. Research suggests that dexmedetomidine can activate the PI3K/AKT pathway, reducing neuroinflammation induced by propofol [18]. Additionally, dexmedetomidine can activate the PI3K/AKT pathway to attenuate neuronal autophagy in traumatic brain injury [19]. Therefore, we associate the dexmedetomidine-induced activation of the PI3K/AKT signaling pathway with the functionality of the brain’s glymphatic system, suggesting it as a potential protective mechanism.

## Results

### Long-term beneficial effects of dexmedetomidine on young mice with multiple sevoflurane exposures

**Fig. 1:**
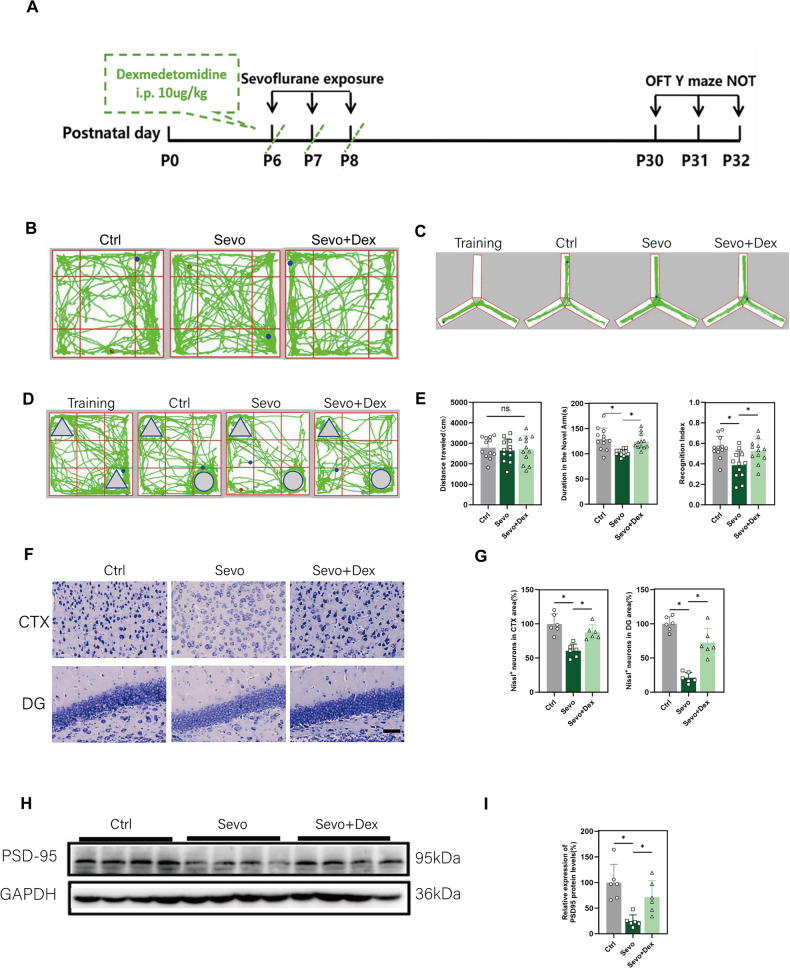
Behavioral tests and neuronal states in P32 mice. **A** Experimental roadmap. **B** Open field test. **C** Y-maze test. **D** Novel object recognition test. **E** Statistical analysis of behavioral tests(n = 12). **F**, **G** Nissl staining and quantitative analysis of hippocampus in P32 mice (n = 6, scale bar = 50 μm). **H**, **I** Western blot and quantitative analysis of PSD95 in hippocampus of P32 mice (n = 6). Data are expressed as the mean ± SD, *p < 0.05; ns not significance, One-way ANOVA and Tukey’s post-hoc test.

Repeated exposure to sevoflurane can lead to long-term behavioral abnormalities in mice, manifested by a decrease of the duration in the novel arms in the Y-maze test and a decrease in the cognitive index in the novel object recognition test (Fig. 1C–E). Compared to the Sevo group, the Sevo+Dex group reversed the Y-maze test’s results (Fig. 1C, E), and an improvement of the cognitive index in the novel object recognition test (Fig. 1D, E). There were no significant differences among the three groups in the open field test (Fig. 1B, E).

To examine the status of neurons, Nissl staining was performed. Compared to the Ctrl group, the Sevo group exhibited a significant decrease in Nissl bodies in the cortical and dentate gyrus (DG) regions of the hippocampal coronal brain sections of P32 mice, along with downregulation of PSD95 expression in the hippocampal tissue (Fig. 1F–I). In contrast, the Sevo+Dex group showed a significant increase in Nissl bodies in the cortical and DG regions, with cells displaying regular morphology and deep purple-blue staining. Additionally, PSD95 expression in the hippocampal tissue was significantly upregulated in the Sevo+Dex group compared to the Sevo group (Fig. 1F–I).

The results from this section indicate that multiple exposures to sevoflurane in young mice can cause long-term neuronal and synaptic damage, subsequently affecting the physiological function of neurons and synapses, leading to cognitive abnormalities in mice. Dexmedetomidine can reduce the extent of neuronal and synaptic damage, alleviating the severity of cognitive dysfunction in mice.

### Dexmedetomidine can attenuate the decline in glymphatic system circulation function induced by sevoflurane in P8 mice

**Fig. 2:**
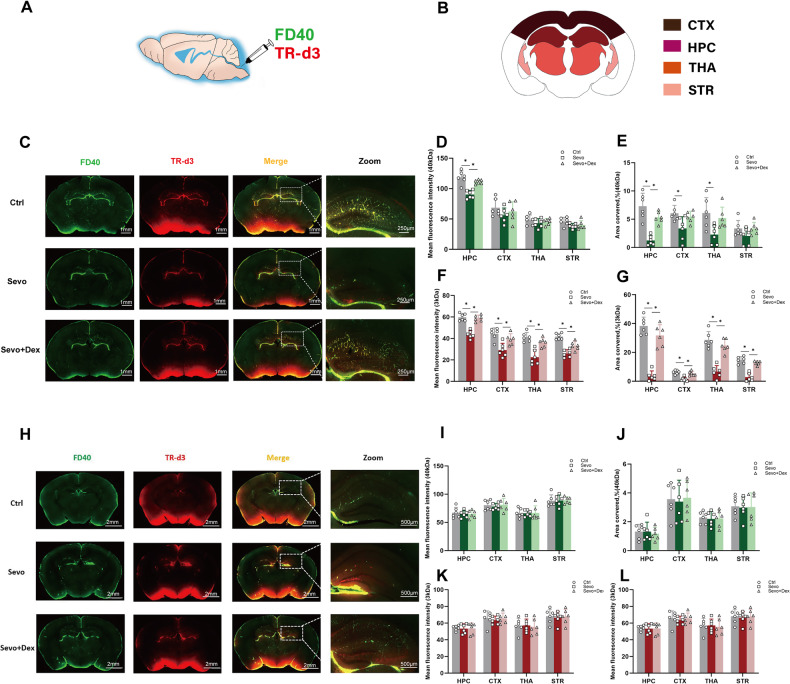
Circulatory function of the glymphatic system in mice. **A** Schematic diagram of tracer injection. **B** Schematic diagram of coronal brain slices in mouse hippocampus. **C** Circulation of tracer FD40 and TR-d3 in P8 mice (n = 6). **D**, **E** The mean fluorescence intensity and distribution area of tracer FD40 in each brain region for P8 mice. **F**, **G** The mean fluorescence intensity and distribution area of tracer TR-d3 in each brain region for P8 mice. **H**, **I** Circulation and quantitative analysis of glymphatic system in P32 mice (n = 6). Data are expressed as the mean ± SD, *p < 0.05. One-way ANOVA and Tukey’s post-hoc test (**D**–**G**, **I**, **K**, **L**); Kruskal-Wallis test and Dunnett test (**J**).

To examine the circulation function of the glymphatic system in mice, the hippocampal coronal brain sections were obtained to measure the mean fluorescence intensity and distribution areas. FITC-d40(40 kDa) and TR-d3(3 kDa) represented metabolic wastes of two molecular weights. Compared to the Ctrl group, the Sevo group exhibited a significant decrease in the distribution area and the average fluorescence intensity of the hippocampus region (Fig. 2C–G). For the TR-d3 tracer in the Sevo group, the distribution area and the average fluorescence intensity were significantly lower than the Ctrl group in the all regions of entire hippocampal coronal brain sections (Fig. 2C, F, G). These results indicate that the circulation capacity of the glymphatic system in the hippocampal tissue of P8 mice significantly declines after multiple exposures to sevoflurane, particularly in the hippocampal region. Compared to the Sevo group, the Sevo + Dex group showed a significant increase in the distribution area and average fluorescence intensity of the hippocampal region in P8 mice (Fig. 2C–G). These results indicate that dexmedetomidine can effectively alleviate the impairment of glymphatic system circulation in the hippocampal tissue of P8 mice induced by multiple exposures to sevoflurane.

Compared to the Ctrl group, there were no significant differences in the average fluorescence intensity and distribution area of P32 mice between the Sevo group and the Sevo + Dex group (Fig. 2H, I). These results indicate that the glymphatic system circulation function in P32 mice exposed to sevoflurane has recovered, and there were no significant differences among the three groups.

### The effect of dexmedetomidine on the AQP4 polarity and quantity of expression in the hippocampal tissue of P8 mice exposed to multiple times of sevoflurane

**Fig. 3:**
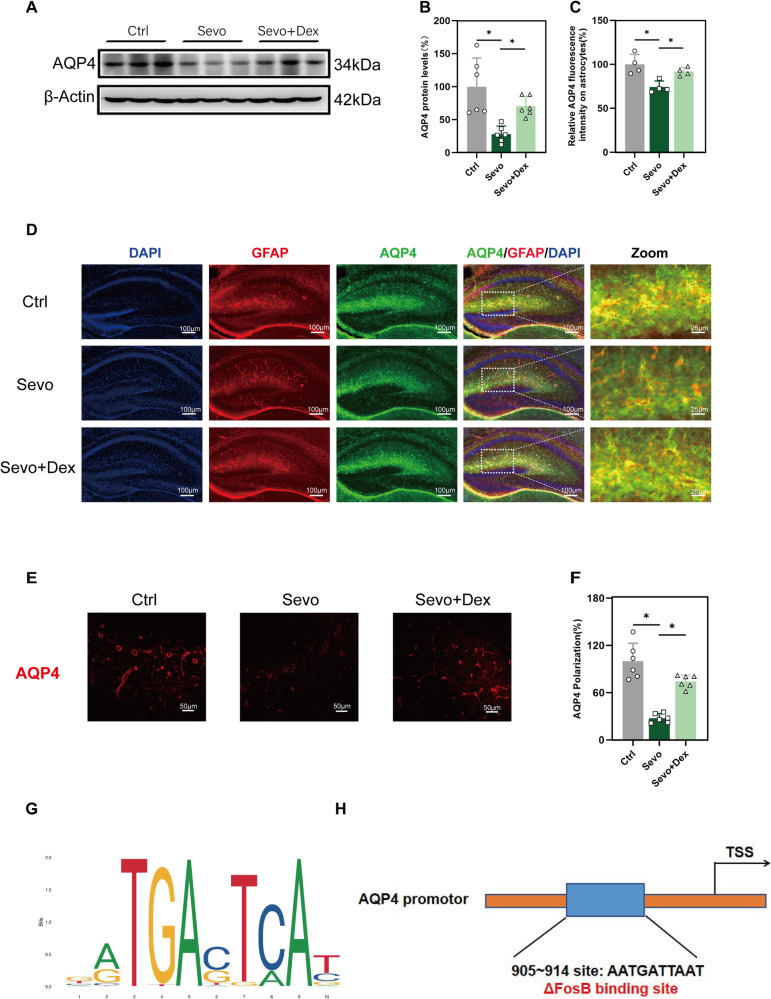
AQP4 polarity and quantity of expression in P8 mice. **A**, **B** Western blot of AQP4 and quantitative analysis in the P8 mouse hippocampus(n = 6). Each experiment was independently repeated three times. **C**, **D** Immunofluorescence and quantitative analysis of AQP4 in the P8 mouse hippocampus(n = 4). **E**, **F** AQP4 polarity and quantitative analysis in the P8 mouse hippocampus(n = 6). **G**, **H** The binding sites of ΔFosB on the AQP4 promoter shown in the diagrammatic drawing. Data are expressed as the mean ± SD, *p < 0.05. One-way ANOVA and Tukey’s post-hoc test.

To further verify the circulating function of the glymphatic system, the expression level and polarity of AQP4 were examined. Compared to the Ctrl group, the Sevo group exhibited a significant decrease both of the protein expression level and the relative fluorescence intensity of AQP4 (Fig. 3A–D). AQP4 polarity in the Sevo group showed a significant decrease (Fig. 3E, F). Compared to the Sevo group, dexmedetomidine attenuated the decline of AQP4 protein level and the AQP4 polarity in the hippocampal tissue of P8 mice (Fig. 3A–F). Then we predicted the potential binding sites of ΔFosB on the AQP4 promoter in the JASPAR database for studying the mechanism further (Fig. 3G, H). These results indicate that dexmedetomidine can effectively alleviate the decrease of the expression level and AQP4 polarity in the hippocampal tissue of P8 mice induced by multiple exposures to sevoflurane. And ΔFosB may be involved in the transcriptional regulation of AQP4.

### The effect of dexmedetomidine on the PI3K/AKT/ΔFosB pathway in the hippocampal tissue of P8 mice exposed to multiple times of sevoflurane

**Fig. 4:**
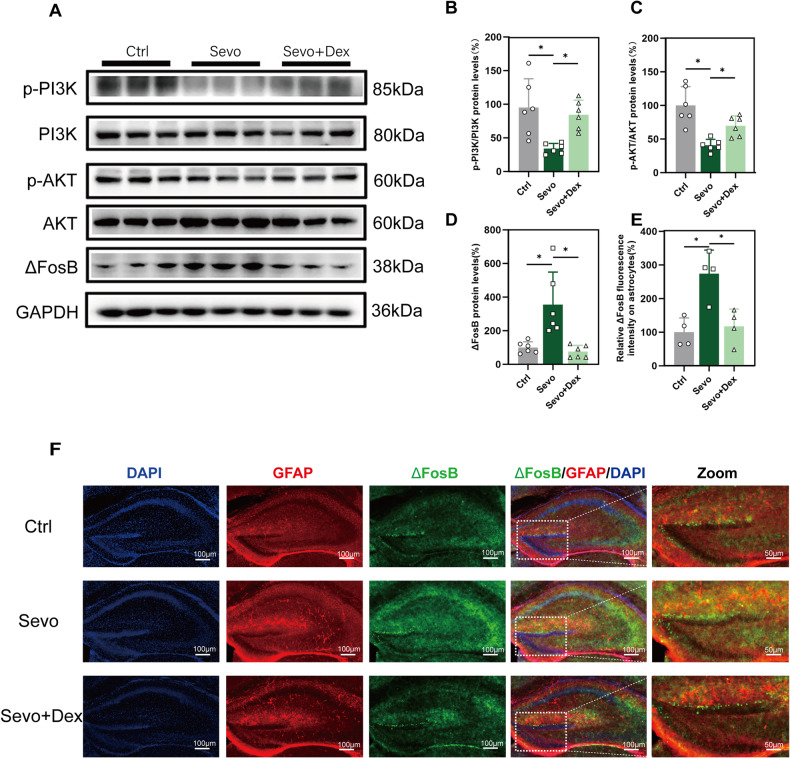
Expression of PI3K/AKT/ΔFosB/AQP4 pathway in P8 mice. **A** Western blot of p-PI3K, PI3K, p-AKT, AKT, and ΔFosB in the P8 mouse hippocampus. **B**–**D** Quantitative analysis of Western blot for P8 mice(n = 6). Each experiment was independently repeated three times. **E**, **F** Immunofluorescence and quantitative analysis of ΔFosB in the P8 mouse hippocampus(n = 4). Data are expressed as the mean ± SD, *p < 0.05. One-way ANOVA and Tukey’s post-hoc test.

To further study the mechanism, we examined hippocampal tissue of P8 mice. Compared to the Ctrl group, the Sevo group exhibited a significant decrease in the protein expression levels of p-PI3K and p-AKT in the hippocampal tissue of P8 mice (Fig. 4A–C). The expression level of ΔFosB protein showed a significant increase (Fig. 4A, D). Immunofluorescence staining results demonstrated co-localization of ΔFosB with GFAP, and the relative fluorescence intensity of ΔFosB was significantly higher in the DG region of the hippocampus (Fig. 4E, F). Compared to the Sevo group, dexmedetomidine attenuated the decline in p-PI3K and p-AKT protein levels, as well as the increase in ΔFosB protein levels in the hippocampal tissue of P8 mice (Fig. 4A–F). These results indicate that dexmedetomidine can alleviate the molecular changes associated with the injury caused by multiple exposures to sevoflurane in young mice.

### The PI3K activator 740Y-P can rescue the extent of AQP4 decrease induced by sevoflurane in primary astrocytes

**Fig. 5:**
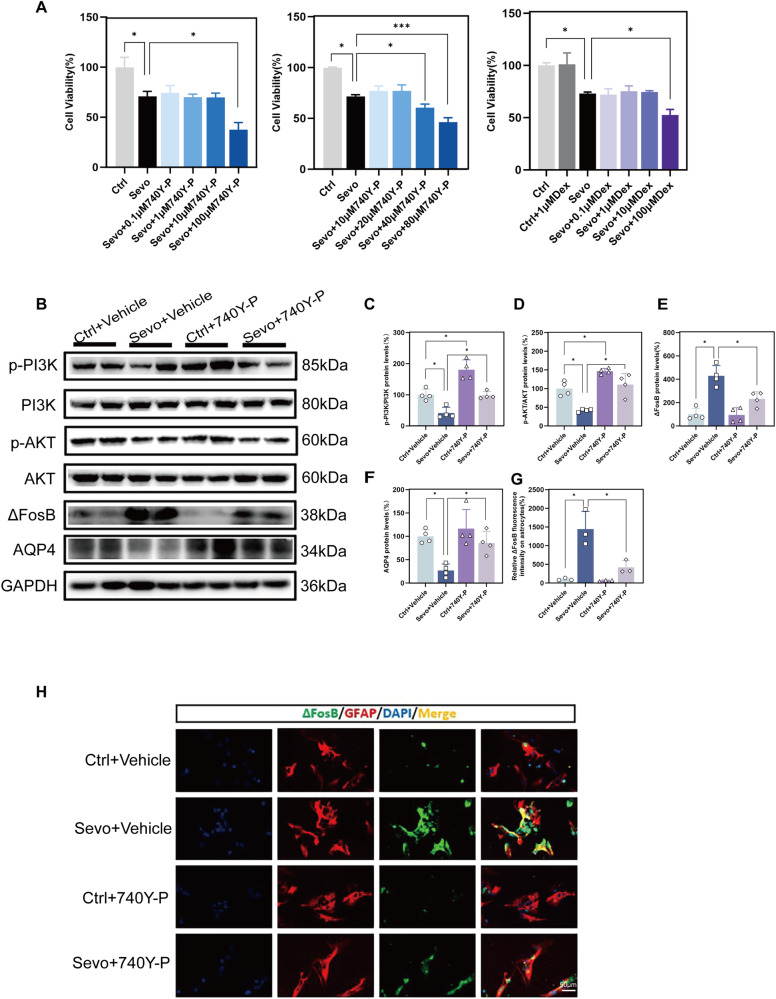
Effect of 740Y-P on the PI3K/AKT/ΔFosB/AQP4 pathway in primary astrocytes. **A** Cell viability of primary astrocytes. **B**–**F** Western blot and quantitative analysis of p-PI3K, PI3K, p-AKT, AKT, ΔFosB, and AQP4 in primary astrocytes(n = 4). **G**, **H** Immunofluorescence and quantitative analysis of ΔFosB in primary astrocytes(n = 3, scale bar = 50 μm). Data are expressed as the mean ± SD, *p < 0.05, ***p < 0.001. One-way ANOVA and Tukey’s post-hoc test.

To determine the regulatory relationship between the PI3K/AKT pathway and its downstream, we used the PI3K agonist 740Y-P. Firstly, we detected the toxic effects of 740Y-P on primary astrocytes, and an appropriate concentration was chosen (Fig. 5A). Before each exposure to sevoflurane, primary astrocytes were treated with 20 μM 740Y-P. After the third exposure to sevoflurane, cells were harvested for relevant experimental assays. Compared to the Ctrl+Vehicle group, the Ctrl + 740Y-P group showed a significant upregulation in the expression levels of p-PI3K/PI3K and p-AKT/AKT (Fig. 5B–D), indicating that 20 μM 740Y-P effectively activates the PI3K/AKT pathway. Compared to the Sevo+Vehicle group, the Sevo+740Y-P group exhibited a significant downregulation in ΔFosB expression levels (Fig. 5B, E, G, H). And as a result, AQP4 expression levels were upregulated (Fig. 5B, F). These experimental results suggest that 740Y-P can alleviate the extent of ΔFosB and AQP4 expression changes induced by sevoflurane, and the PI3K/AKT pathway is involved in regulating the expression of ΔFosB and AQP4 proteins in primary astrocytes.

### Transfection of siFosB can alleviate the extent of AQP4 decrease induced by sevoflurane in primary astrocytes

**Fig. 6:**
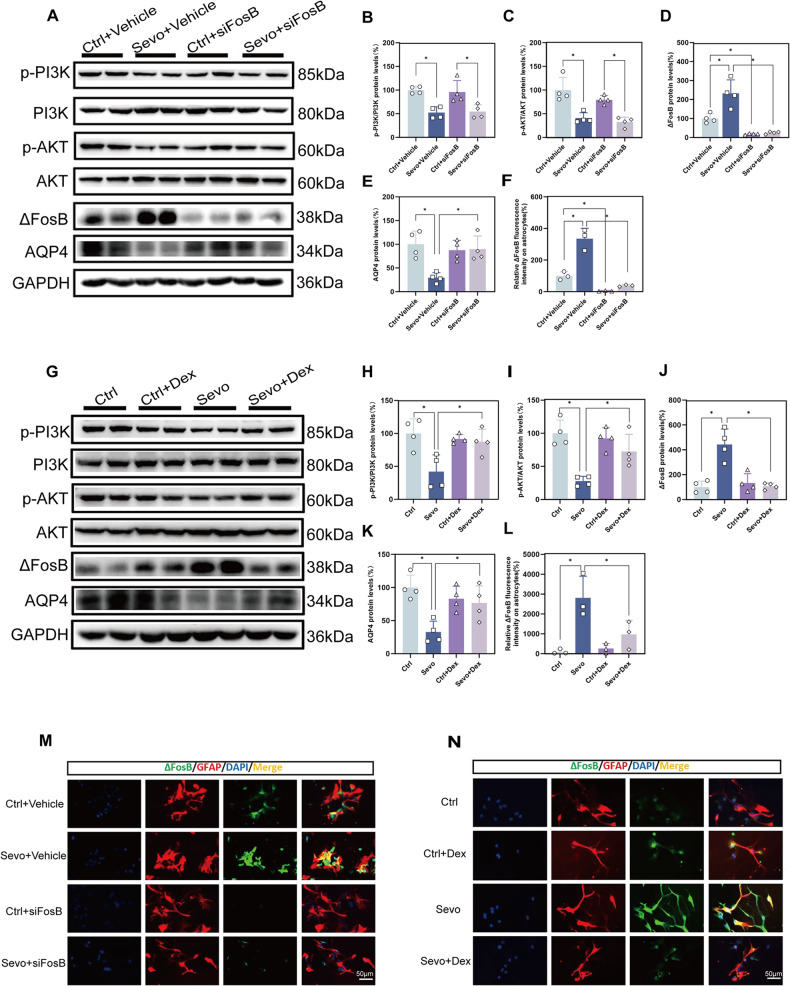
Effects of siFosB and dexmedetomidine on the PI3K/AKT/ΔFosB/AQP4 pathway in primary astrocytes. **A**–**E** Western blot and quantitative analysis of p-PI3K, PI3K, p-AKT, AKT, ΔFosB, and AQP4 in primary astrocytes treated with siFosB(n = 4). **G**–**K** Western blot and quantitative analysis of p-PI3K, PI3K, p-AKT, AKT, ΔFosB, and AQP4 in primary astrocytes treated with dexmedetomidine(n = 4). **F**, **M** Immunofluorescence and quantitative analysis of ΔFosB in primary astrocytes treated with siFosB(n = 3, scale bar = 50 μm). **L**, **N** Immunofluorescence and quantitative analysis of ΔFosB in primary astrocytes treated with dexmedetomidine(n = 3, scale bar = 50 μm). Data are expressed as the mean ± SD, *p < 0.05. One-way ANOVA and Tukey’s post-hoc test.

To explore the regulatory relationship between ΔFosB and AQP4, we used small interfering RNA technology. Prior to the first exposure to sevoflurane, primary astrocytes were transfected with siFosB. After the third day of sevoflurane exposure, cells were harvested for relevant experimental assays. Compared to the Ctrl + Vehicle group, the Ctrl + siFosB group showed a significant downregulation in ΔFosB expression levels (Fig. 6A, D, F, M), indicating that siFosB transfection effectively silenced ΔFosB expression. Compared to the Sevo + Vehicle group, the Sevo+siFosB group exhibited a significant downregulation in ΔFosB expression levels (Fig. 6A, D, F, M), and AQP4 expression levels were significantly increased (Fig. 6A, E). These experimental results suggest that transfection of siFosB prior to exposure to sevoflurane can alleviate the extent of AQP4 decrease in primary astrocytes, indicating a negative regulatory role of ΔFosB on AQP4.

### The effect of dexmedetomidine on the PI3K/AKT/ΔFosB/AQP4 pathway in primary astrocytes

To further verify the molecular mechanism of the protective effect of dexmedetomidine, we further demonstrated that using primary astrocyte experiments. Compared to the Sevo group, the Sevo+Dex group showed a significant increase in the expression levels of p-PI3K/PI3K and p-AKT/AKT (Fig. 6G–I), a significant downregulation in ΔFosB expression levels (Fig. 6G, J, L, N), and as a result, AQP4 expression levels were upregulated (Fig. 6G, K). These experimental results suggest that dexmedetomidine can attenuate the extent of AQP4 decrease by activating the PI3K/AKT/ΔFosB/AQP4 pathway.

## Discussion

**Fig. 7:**
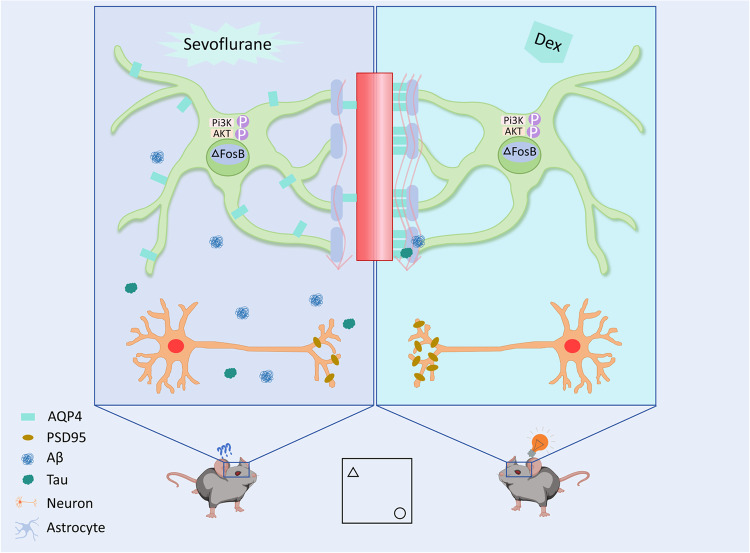
Schematic drawing of the major finding. Sevoflurane inhibits the activation of PI3K/AKT pathway and up-regulates the expression of transcription factor ΔFosB. ΔFosB binds to the AQP4 promoter region, represses AQP4 transcription, and down-regulates its expression. The AQP4 polarity and its expression are down-regulated, resulting in a decrease in the circulation function of the glymphatic system. The accumulation of metabolic waste products in the brain damages neurons and synapses, leading to a long-term decline in learning and memory ability in young mice. Dexmedetomidine alleviates sevoflurane-induced glymphatic dysfunction through PI3K/AKT/ΔFosB/AQP4 pathway.

In our study, we have discovered for the first time that multiple exposures of sevoflurane during the developmental period of mice can lead to a decrease in their glymphatic system circulation and induce long-term impairments in learning and memory abilities. This provides a novel perspective to elucidate the mechanisms underlying sevoflurane-induced brain injury during the developmental period (Fig. 7).

The glymphatic system is a clearance system for intracranial metabolic waste, which facilitates the efficient elimination of soluble proteins and metabolites in the central nervous system through a unique perivascular space formed by astrocytes [7, 20]. We chose TR-d3(MW 3kD) and FD40(MW 40kD) as tracers to simulate the circulation of Aβ1-42(MW 4kD) and p-Tau(PHF1, MW 55kD), for they have similar molecular weights, respectively. We found that among all brain regions, hippocampal region displayed the most severe decline in glymphatic system circulation for both tracers. Furthermore, the hippocampus region is closely related to cognitive functions [21]. Therefore, we selected this very region as the subject of our study.

Aβ [6, 22]and Tau [23, 24] in the brain have been shown to rely on the glymphatic system pathway for metabolism. We found that as the circulating capacity of the glymphatic system decreased with sevoflurane, the expression of Aβ1-42 and p-Tau(PHF1,Ser396/404) increased in the hippocampal tissue of P8 mice (Supplementary Fig. 1A–D). Dexmedetomidine rescued the decline in glymphatic system circulating capacity in P8 mice and alleviated the extent of Aβ1-42 and p-Tau(PHF1,Ser396/404) increase (Supplementary Fig. 1A–D). This indicates that the circulating capacity of the glymphatic system decreased with sevoflurane anesthesia, which leads to the accumulation of Aβ1-42 and phosphorylated Tau in the hippocampus of P8 mice.

Aβ and Tau impair the structure and function of neurons and synapses through multiple pathways [25–29]. Additionally, both Aβ and Tau are toxic to the postsynaptic scaffolding protein PSD95 [29–32]. Immunoprecipitation have demonstrated Aβ interacts with PSD95 at specific sites [33] and it has targeted attack on PSD95 [34]. As a result, we detected down-regulated PSD95 expression in hippocampal tissue of P8 mice in sevoflurane group(Supplementary Fig. 1A–B). PSD95 plays a crucial role in maintaining synaptic plasticity and regulating learning and memory [35, 36]. Studies from PSD95-knockout mice exhibited severe synaptic dysfunction and behavioral abnormalities at P35 [37], which is consistent with our research findings. We found that PSD95 expression was down-regulated of P32 mice in sevoflurane group, which damaged to the learning and memory abilities. Xia and colleagues [38] also came to a similar conclusion through fear conditioning test and observation of the synaptic ultrastructure by electron microscopy, when the young mice were repeatedly exposed to sevoflurane. This indicates that the persistent decline of PSD95 and a decrease in the number of Nissl bodies jointly lead to decreased learning and memory abilities of P32 mice in sevoflurane group. However, as the circulating capacity of the glymphatic system recovers in the sevoflurane group of P32 mice, the expression levels of Aβ1-42 and phosphorylated Tau returned to normal(Supplementary Fig. 1F, G). The learning and memory abilities of P32 mice in Sevo group were still impaired. This indicates that the damage to synapses and neurons caused by metabolic wastes cannot be reversed.

We simulated a model of repeated exposure to sevoflurane through in vivo and in vitro experiments. In our study, we found that the phosphorylation expression of the PI3K/AKT pathway was inhibited, which is similar to the findings of Zhong and colleagues [39]. Additionally, we discovered for the first time that the expression of ΔFosB in the hippocampal tissue of mice exposed to sevoflurane anaesthesia multiple times acutely increased. ΔFosB, encoded by the *fosB* gene, is a member of the Fos transcription factor family. These Fos family proteins form heterodimers with Jun family proteins, constituting the active AP-1 transcription factor [11, 40, 41]. AP-1 is an important downstream target of AKT [42], and further confirmation of the regulation of ΔFosB by the PI3K/AKT pathway was demonstrated by treating primary astrocytes with the PI3K agonist 740Y-P. Simultaneously, transfection of primary astrocytes with siFosB resulted in upregulation of AQP4 expression, further confirming the negative regulatory relationship between ΔFosB and AQP4. The clearance capability of the glymphatic system is closely related to AQP4 highly polarized on the endfeet of astrocytes [43]. We found that after multiple exposures to sevoflurane, AQP4 expression and its polarity in P8 mice was down-regulated, further confirming the decreased circulation capability of the glymphatic system. Although Gao et al.’s [44] found that AQP4 expression was upregulated in primary astrocytes after a single sevoflurane treatment. The difference in regulations may be related to the frequency of sevoflurane exposures, as multiple exposures to sevoflurane are much more damaging than single exposure to the brain [45]. Therefore, sevoflurane can damage or protect the brain under different conditions. In addition, we observed reactive astrogliosis in terms of morphology with P8 mice exposed to sevoflurane by Sholl analysis(Supplementary Fig. 1H, I). However, at P32, we did not observe morphological differences about astrocytes among the three groups(Supplementary Fig. 1J). This is because in mild to moderate cases of reactive astrogliosis, if the triggering mechanisms have been resolved, the astrocytes revert to an appearance like healthy tissue [46]. What is more, previous studies have shown that impaired glymphatic system circulation occurs when reactive astrogliosis is present [47], as the polarized distribution of AQP4 is closely associated with reactive astrogliosis [48, 49]. Hence, this further supports the evidence of downregulation in the glymphatic system circulation in P8 mice exposed to sevoflurane and the ameliorating effect of dexmedetomidine on the injury.

Previous studies have shown that dexmedetomidine can alleviate the increase in Tau protein phosphorylation induced by sevoflurane in young mice by activating α2 receptors [50], and it can also mitigate the damage to mitochondria caused by sevoflurane [51]. We found that dexmedetomidine increased the phosphorylation expression of PI3K/AKT induced by sevoflurane in P8 mice, reversed the acute elevation of ΔFosB, increased AQP4 expression, and rescued the circulatory function of the glymphatic system. In long-term assessments, mice in the dexmedetomidine group exhibited better learning and memory abilities than those in the sevoflurane group. This indicates that dexmedetomidine can protect against sevoflurane-induced damage to the circulatory function of the glymphatic system through the PI3K/AKT/ΔFosB/AQP4 signaling pathway on astrocytes. This provides a novel perspective for explaining the mechanism of dexmedetomidine for brain protection.

The impact of general anesthetics on the function of the glymphatic system has always been a subject of debate. Previous studies show that different types of general anesthetics [9] and varying concentrations of inhaled anesthetics [52] can have different effects, either positive or negative, on the circulation function of the glymphatic system. In previous studies, a mixture of ketamine and xylazine helped to mimic natural sleep processes, which is beneficial for the circulation of the glymphatic system [53]. Consequently, ketamine and xylazine are often used as a paradigm in animal anesthesia models for glymphatic system research [9, 43], which is also applied in our study. It has been reported that isoflurane (maintained at 1–2% for 30 min) can reduce the circulatory function of the glymphatic system in mice compared with a mixture of ketamine and xylazine [9]. Therefore, when studying the function of the glymphatic system, we cannot ignore the potential effects of anesthetics on the circulatory capacity of the glymphatic system.

This study still has certain limitations. We do not know when the glymphatic system restores normal circulatory function. Meanwhile, we do not investigate the underlying mechanism of the dexmedetomidine about α2 receptor.In future work, we will further focus on α2 receptors and the interactions between astrocytes and neurons.

The above experiments demonstrate that repeated exposure to sevoflurane can lead to a downregulation of the circulatory function of glymphatic system and a decline in long-term learning and memory abilities in young mice. Dexmedetomidine, through its action on the PI3K/AKT/ΔFosB/AQP4 pathway, provides long-term brain protection during the developmental period.

## Materials/subjects and methods

### Mice, anesthesia, and treatment

This experiment used C57BL/6 mice, and all experimental protocols were approved by the Animal Care and Use Committee of China Medical University, with the approval number CMU2020307, and conducted in accordance with the “Regulations on the Management of Experimental Animals in China.” Every effort was made to minimize the number of animals used and to reduce their pain and suffering. The manuscript was written according to ARRIVE.

All the animals were randomly grouped for experiments. Based on previous experiments [54], P6 mice were exposed to inhalation for 2 h per day for 3 consecutive days. The experimental groups were as follows: (1) Ctrl group: intraperitoneal injection of normal saline, followed by inhalation of 40% oxygen after 30 min. (2) Sevo group: intraperitoneal injection of normal saline at a dose of 10 μg kg^−1^, followed by inhalation of 3% sevoflurane and 40% oxygen after 30 min. (3) Sevo + Dex group: intraperitoneal injection of dexmedetomidine at a dose of 10 μg kg^−1^ [50], followed by inhalation of 3% sevoflurane and 40% oxygen after 30 min.

Gas monitoring and control were performed using a monitoring device (Datex-Ohmeda S/5, US) and a sevoflurane vaporizer (Dräger Vapor 2000, Germany). Additionally, a heating blanket was used to maintain the rectal temperature of the young mice at 37 ± 0.5 °C.

### Primary astrocytes, anesthesia, and treatment

As previously described [55], primary astrocytes were harvested and cultured from newborn mice, and randomly divided into the following groups: (1) Ctrl + Vehicle (2) Sevo + Vehicle (3) Ctrl + Dex (4) Sevo + Dex (5) Ctrl + 740Y-P (6) Sevo + 740Y-P (7) Ctrl + siFosB (8) Sevo+siFosB. The control group was exposed to 21% oxygen and 5% carbon dioxide, while the sevoflurane group was exposed to 3% sevoflurane combined with 21% oxygen and 5% carbon dioxide. Primary astrocytes were pretreated with the PI3K activator 740Y-P (HY-P0175, MCE, US) at a concentration of 20 μM for 1 h before sevoflurane exposure, with the corresponding dose of DMSO given to the control group. Based on previous research and cell viability test results, primary astrocytes were treated with 1 μM dexmedetomidine for 30 min before sevoflurane exposure [51, 56]. When the astrocytes purity reached 95% or above, the anesthesia began on the 14th day of astrocytes culture. Each exposure lasted for 2 h, and this protocol was repeated for 3 consecutive days [57].

### Behavioral tests

The investigator was blinded to the group allocation during the behavioral tests.

#### (1) Open field test(OFT)

OFT is a method used to evaluate the autonomous behaviors, exploratory behavior and anxiety level of experimental animals in a novel environment. The open field apparatus is a topless rectangular container measuring 40 cm × 40 cm × 30 cm. The central 20 cm × 20 cm area is designated as the center zone, while the remaining area is considered the periphery. Following the methods described in previous studies [58], mice were gently placed in one corner of the open field and allowed to freely explore for 10 min. During this time, data and trajectories were recorded using the SMART™ (San Diego Instruments, San Diego, CA, USA) tracking and analysis system. For mice, spending less time in the center zone indicates increased anxiety-like behavior [58].

#### (2) Y-maze test

The Y-maze test is a behavioral experiment used to assess spatial memory in mice. The Y-maze consists of a central triangular chamber and three equally sized radial arms (30 cm × 15 cm × 6 cm), with each arm positioned at a 120° angle to each other. The arms are labeled as the initial arm, familiar arm, and novel arm. Distinctive visual cues in the form of different shapes and colors are placed at the distal ends of each arm to assist the mice in navigating and distinguishing between the arms. Following previous research methods [59], the experiment is divided into two parts. In the learning phase, the novel arm is closed off. The mouse is gently placed in the initial arm of the Y-maze and allowed to freely explore the other two arms for 10 min. Then the mouse is given a rest period of 1 h. The second part is the testing phase. All three arms of the Y-maze are open, and the mouse is allowed to freely explore all three arms for 2 min while the SMART™ tracking system is activated to collect data and track the trajectory. The number of entries and the time spent in each arm are recorded.

#### (3) Novel object recognition test(NOT)

NOT is a behavioral experiment used to assess learning and memory abilities in mice by taking advantage of their inherent tendency to explore novel objects. Following previous experiments [60, 61], this experiment is divided into two parts. In the learning phase, two identical triangular objects are placed diagonally in zones of the open field. The mouse is gently placed and allowed to explore for 10 min. Then the mouse is given a rest period of 1 h. The second part is the testing phase. One of the triangular objects is replaced with a new cylindrical object. The mouse is allowed to freely explore for 5 min. The SMART™ tracking system is activated to collect data and track the trajectory. The time spent by the mouse in the old object and the novel object is recorded separately. The recognition index [60], calculated as the time spent with the novel object divided by the sum of the time spent with the novel and old objects, is used to evaluate the mouse’s short-term working memory.

### Tracer injection

The investigator was blinded to the group allocation during the tracer injection.

Mice were anesthetized using ketamine at a dose of 0.12 mg/g and xylazine at a dose of 0.01 mg/g [6, 9, 53]. After exposing the atlanto-occipital membrane, a 30G needle was used to puncture the membrane, and the needle was secured. A microinjection pump was connected, and the tracer solution composed of FITC-d40(D1845, ThermoFisher) and TR-d3(D3328, ThermoFisher) at a concentration of 1% was prepared. According to previous studies [12], for P8 mice, 1.5 μl of the tracer solution was injected, while for P32 mice, 10 μl was injected. The injection was carried out at a constant rate over a duration of 10 min. After the tracer injection, the tracer was allowed to circulate for 30 min before the mouse undergoing transcardial perfusion with 4% paraformaldehyde. The brains were then extracted and sliced into 100 μm-thick coronal sections using a vibrating microtome(VT1000S, Leica, Germany). Imaging was performed by Zeiss Axio Scan.Z1 confocal microscope system (Zeiss, Jena, Germany).

To quantify tracer movement into different regions, slice images were analyzed in Fiji/Image J(National Institutes of Health) as described previously [6, 12]. For each slice, color channels were split. The partitioning of brain regions was done by superimposing the anatomical map on another layer of Fiji/Image J(National Institutes of Health) (Supplementary Fig. 1K). The mean fluorescence intensity was measured for the regions of interest (ROI), including hippocampus, cortex, thalamus and striatum. The mean fluorescence intensity was calculated by the threshold at a pixel intensity of 60 (out of 255). And the thresholded area expressed as a % of overall slice area.

### AQP4 polarization

As the previous methods [62, 63], the median immunofluorescence intensity of perivascular regions was measured to measure perivascular AQP4 polarization. In short, a threshold analysis measured the % of the region exhibiting AQP4 immunofluorescence greater than or equal to perivascular AQP4 immofluorescence (AQP4% Area). “Polarization” = 100 − AQP4% Area. Hence AQP4 polarization is a relative measurement of AQP4 localization: increasing polarization indicating higher perivascular AQP4-immunoreactivity relative to lower parenchymal AQP4-immunoreactivity, whereas reduced polarization indicating lower perivascular AQP4-immunoreactivity relative to higher parenchymal AQP4-immunoreactivity.

### Bioinformatic analysis

JASPAR database (http://jaspar.genereg.net/) was employed to predict the binding relationship between the predicted target transcription factor (TF) and AQP4. Firstly, the 5’ end sequence extending from the transcription start site (TSS) region at 2000 bp in the upstream of AQP4 gene was downloaded from National Center of Biotechnology Information (NCBI) database (https://www.ncbi.nlm.nih.gov/). Thereafter, this sequence was compared with the TF in JASPAR database with a perfect match >80%. The result was determined as the possible binding site on the TF.

### Nissl staining

Paraffin sections were subjected to routine deparaffinization and hydration, followed by three washes with distilled water for 5 min each. The sections were then stained with a 0.1% toluidine blue solution (Beyotime Biotechnology, China) and incubated in a temperature-controlled oven at 50–60°C for 40 min. After staining, the sections were washed three times with distilled water for 5 min each. Decoloration was carried out using a gradient of ethanol concentrations, followed by clearing in xylene. Imaging was performed by Zeiss Axio Scan.Z1 confocal microscope system (Zeiss, Jena, Germany). Fiji/Image J(National Institutes of Health) was used to quantify the Nissl^+^ neurons. The mean optical density was measured for the regions of interest (ROI) and calculated by the threshold.

### Western Blot

The proteins from mice hippocampus and primary astrocytes were extracted by Western-IP Lysis Buffer (Beyotime, China) and quantified by BCA protein assay kit (Beyotime, China). In total, 10 μg of total proteins were electrophoresed on 12% SDS polyacrylamide gels and transferred onto nitrocellulose membranes. The membranes were blocked with 5% non-fat milk for 90 min at room temperature. Membranes were incubated with primary antibodies overnight at 4 °C as the following: rabbit anti-PI3K (AF6241,1:1000,Affinity), rabbit anti-phospho-PI3K at Tyr607(AF3241,1:1000,Affinity), rabbit anti-AKT(YT0185,1:500,Immunoway), mouse anti-phospho-AKT at Ser473 (YP0006,1:2000,Immunoway),rabbit anti-FosB antibody (ab184938,1:1000,Abcam), rabbit anti-AQP4 antibody (16473-1-AP,1:1000,Proteintech), rabbit anti-PSD95 antibody (YT3879,1:2000,Immunoway), rabbit anti-PHF1 antibody (15663-1-AP,1:1000, Proteintech), mouse anti-β-actin antibody (TA-09,1:5000,Amresco) and mouse anti-GAPDH antibody (TA-08,1:5000,Amresco). Fiji/Image J(National Institutes of Health) was used to calculate and quantify the gray values.

### Immunofluorescence staining

Mice brains were removed and postfixed with 4% PFA. Parts of the brains were sliced into 100 μm-thick using a vibrating microtome(VT1000S, Leica, Germany). These sections were used to study AQP4 polarity. The other brains were gradient dehydration in sucrose solution. And then tissues were embedded in OCT compound, and cryosectioned at 30 μm (Leica CM1900, Germany) for immunofluorescence study. The primary antibodies were used as follows:rabbit anti-FosB antibody(ab184938,1:500,Abcam), mouse anti-GFAP antibody(sc-33673,1:400,Santa Cruz), rabbit anti-AQP4 antibody(16473-1-AP,1:500,Proteintech).Alexa Fluor 594 donkey anti-mouse (1:500, Invitrogen, A21203) and Alexa Fluor 488 donkey anti-rabbit (1:500, Life technology, A21206) served as the secondary antibody. Imaging was performed by Zeiss Axio Scan.Z1 confocal microscope system (Zeiss, Jena, Germany). Fluorescent signals were examined using a laser scanning confocal microscopy(TC SP8, Leica, Germany) to detect the AQP4 polarity. Fiji/ Image J(National Institutes of Health) was used to quantify the mean fluorescence intensity. For each slice, color channels were split. The mean fluorescence intensity was measured for the regions of interest (ROI) and calculated by the threshold.

### Sholl analysis

According to the previous method [64, 65], the image stack of fluorescent astrocytes was tracked and reconstructed by threshold using Fiji/ImageJ(National Institutes of Health), which was represented by two-dimensional binarization. In short, we used the Sholl method of concentric circles. We analyzed each astrocyte by selecting its center in the ROI region and then running the Sholl analysis program. The program counted the number of intersections on every circle.

### Cell viability assay

Primary astrocytes were seeded into a 96-well plate and allowed to adhere, and then the corresponding treatment factors were added. After discarding the culture medium, serum-free medium containing 20 μl of CellTiter 96 AQueous One Solution Reagent(Promega, USA) was added to each well. The plate was incubated at 37 °C with 5% CO2 for 3 h. The optical density (OD) was measured at 490 nm using a microplate reader.

### Enzyme-linked immunosorbent assay (ELISA) analysis

Aβ1-42(KMB3441, Thermo Fisher Scientific) ELISA kit was performed according to the manufacturer’s instructions. Hippocampus was homogenized in 50 μl of Tris-HCl 50 mM (pH 8.0) containing 5 M guanidine-HCl, and incubated for 4 h at room temperature. Samples were then diluted ten-fold with cold PBS with 1X protease inhibitor cocktail. After dilution, samples were centrifuged at 16,000 × *g* for 20 min at 4 °C. The supernatant was collected for ELISA. Ninety-six–well plates were read at 450 nm using a microplate reader (ELX808, Bio-Tek, USA).

### siRNA transfection

Primary astrocytes were seeded into a 6-well plate and allowed to grow to 30–40% confluency before performing plasmid transfection. The siRNA was diluted in DEPC-treated water at a 50-fold dilution. Lipofectamine 3000(ThermoFisher,USA) and siRNA were separately added to serum-containing medium and allowed to incubate for 10 min. The transfection mixture was then added to each well of the 6-well plate, with a volume of 100 μl per well.

### Statistical analysis

Based on the previous studies [54], sufficient power to detect a significant effect should be achieved using 12–20 mice per group for behavioral experiments, four to six mice or cell (primary astrocyte) samples per group for the western blot, and ELISA analyses, and three to six mice or cell(primary astrocyte) samples per group for the immunostaining studies.

All the experimental assays in this study were repeated, and the data are presented as mean ± standard deviation. GraphPad Prism 8.0 and IBM SPSS 26 were used for the statistical analyses. For comparisons of means in samples with normal distributions and homogeneous variances, one-way ANOVA was used to compare the means among multiple groups, followed by Tukey’s *post-hoc* test. In cases of a non-normal distribution (Shapiro-Wilk test) or unequal variances (Brown-Forsythe test), a nonparametric Kruskal-Wallis test was used for comparisons between more than two means, followed by Dunnett test. A *p* < 0.05 was considered statistically significant.

### Supplementary information

Supplementary materials

Original images of western blot

## Data Availability

The datasets generated during or analyzed during the current study are available from the corresponding author upon reasonable request.
