# Supplementary material for: Dexmedetomidine improves the circulatory dysfunction of the glymphatic system induced by sevoflurane through the PI3K/AKT/ΔFosB/AQP4 pathway in young mice
Source: Cell Death Dis. 2024 Jun 25;15(6):448. doi: 10.1038/s41419-024-06845-w (PMC11199640; doi:10.1038/s41419-024-06845-w)
Supplement: Supplementary file 1 — Supplementary materials [file 41419_2024_6845_MOESM1_ESM.pdf]

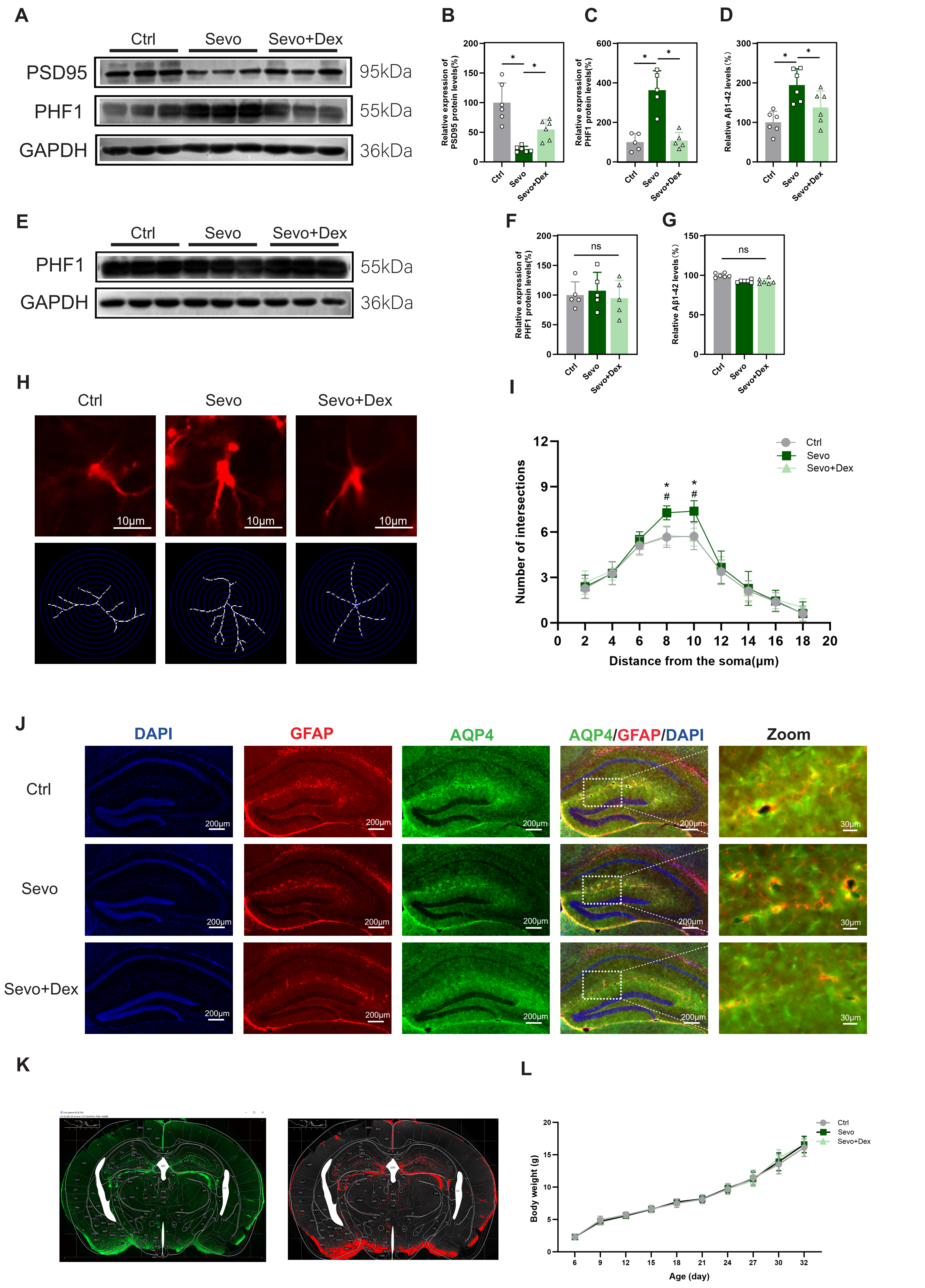
A
Ctrl
Sevo
Sevo+Dex
PSD95
95kDa
PHF1
55kDa
GAPDH
36kDa
B
Relative expression of PSD95 protein levels(%)
C
Relative expression of PHF1 protein levels(%)
D
Relative Aβ1-42 levels (%)
E
F
ns
G
H
10μm
I
Number of intersections
Distance from the soma(μm)
J
DAPI
GFAP
AQP4
AQP4/GFAP/DAPI
Zoom
200μm
30μm
K
L
Body weight (g)
Age (day)

Supplementary figure legends

A-C. Western blot and quantitative analysis of PSD95 and p-Tau(PHF1, Ser396/404) in hippocampus of P8 mice (n=6).

D. Quantitative analysis of Aβ1-42 for P8 mice hippocampus by ELISA(n=6).

E-F. Western blot and quantitative analysis of p-Tau(PHF1, Ser396/404) in hippocampus of P32 mice (n=6).

G. Quantitative analysis of Aβ1-42 for P32 mice hippocampus by ELISA(n=6).

H. Representative images of astrocytes for the Sholl analysis.

I. Quantification of the number of intersections in the sholl analysis(n=6).

J. Immunofluorescence of GFAP in the P32 mouse hippocampus(n=4).

K. Schematic of brain region for tracers and calculation mode diagram.

L. Body weight.

*$p<0.05$,compared with Ctrl group; $^{\#}p<0.05$,compared with Sevo+Dex group; ns, not significance. One-way ANOVA and Tukey's post-hoc test(A-G,J,L); Kruskal-Wallis test and Dunnett test(H-I).

Table S1. Blood gas analysis of P8 mice

Blood Gas Analysis	Ctrl	Sevo	Sevo+ Dex	P value
pH	7.31±0.02	7.32±0.03	7.33±0.03	0.383
PaCO2	37.53±2.08	36.28±1.95	35.85±2.28	0.353
PaO2	151.00±8.02	156.9±7.46	153.78±5.84	0.351
