# Supplementary figures and images for: Dexmedetomidine improves the circulatory dysfunction of the glymphatic system induced by sevoflurane through the PI3K/AKT/ΔFosB/AQP4 pathway in young mice
Source: Cell Death Dis. 2024 Jun 25;15(6):448. doi: 10.1038/s41419-024-06845-w (PMC11199640; doi:10.1038/s41419-024-06845-w)

Fig.1H

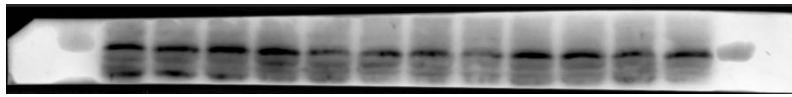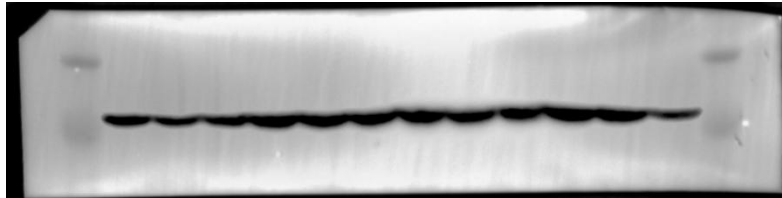

Fig.3

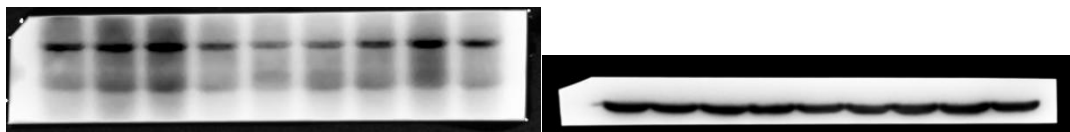

Fig.4

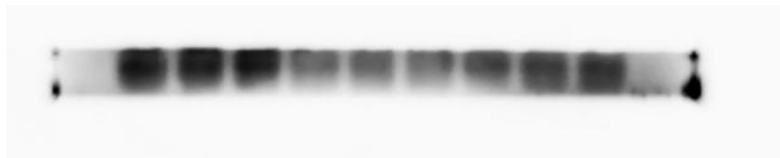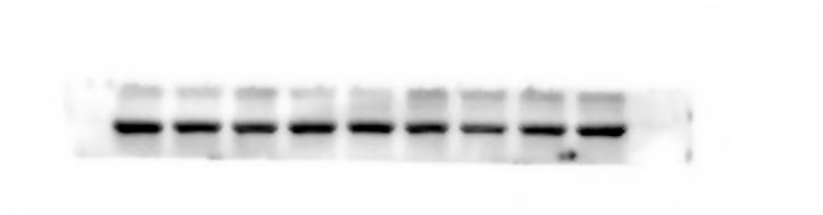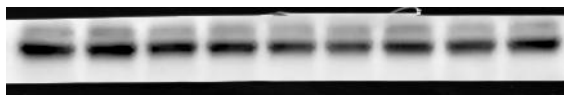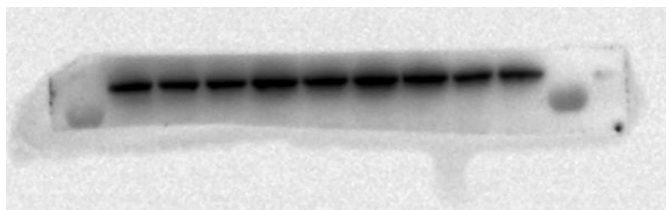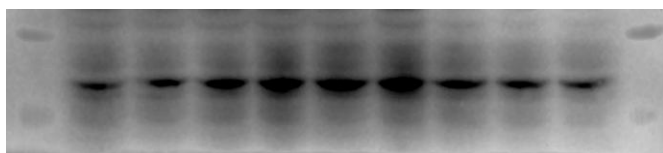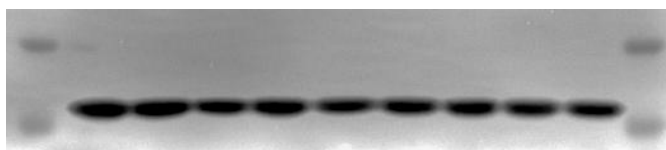

**Fig.5**

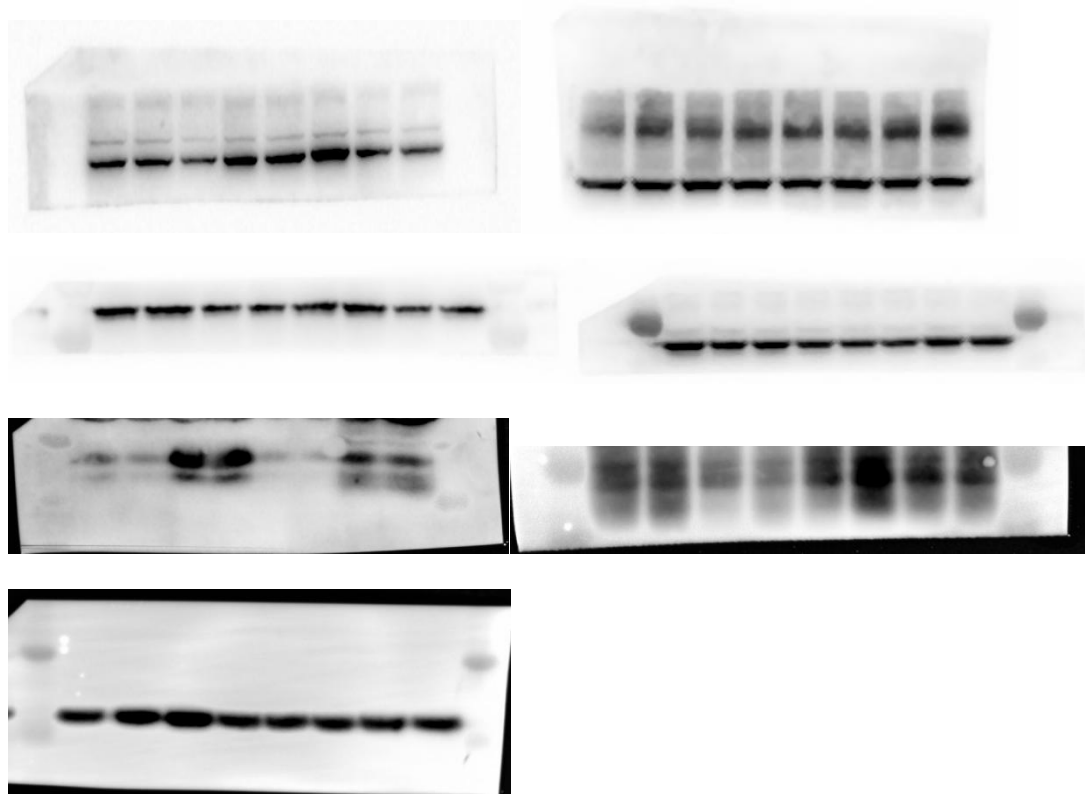

**Fig.6**

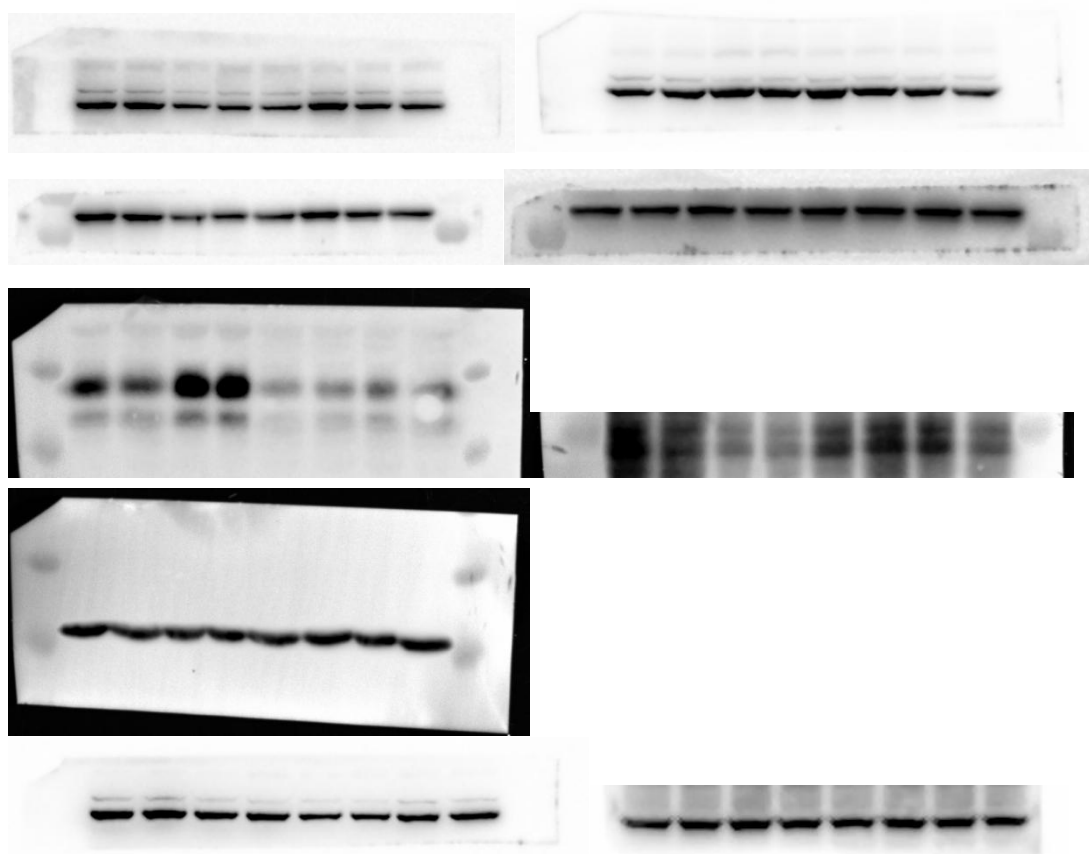

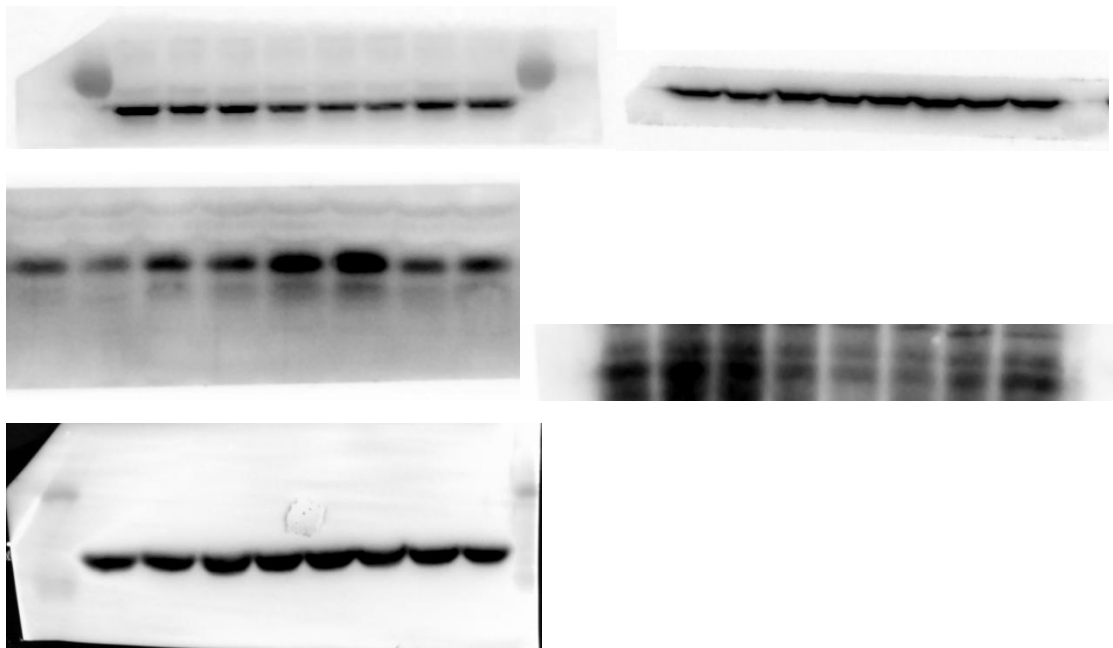

**Supplementary figure 1**

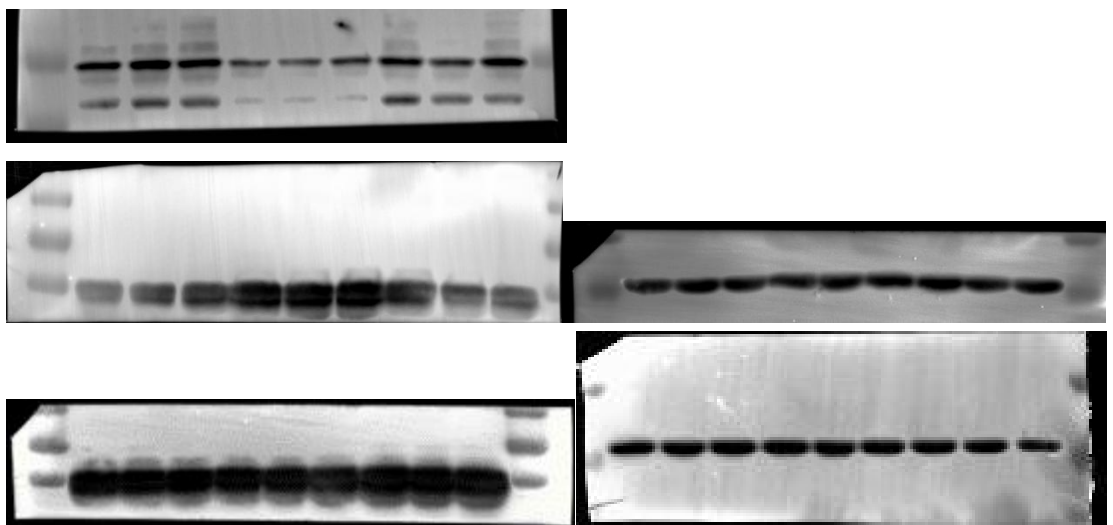

Supplement: Supplementary file 2 — Original images of western blot [file 41419_2024_6845_MOESM2_ESM.pdf]
